# Life expectancy difference and life expectancy ratio: two measures of treatment effects in randomised trials with non-proportional hazards

**DOI:** 10.1136/bmj.j2250

**Published:** 2017-05-25

**Authors:** Hakim-Moulay Dehbi, Patrick Royston, Allan Hackshaw

**Affiliations:** 1 Cancer Research UK and UCL Cancer Trials Centre, 90 Tottenham Court Road, London, W1T 4TJ, UK; 2 MRC Clinical Trials Unit at UCL, Aviation House, 125 Kingsway Road, London, WC2B 6NH, UK

## Abstract

The hazard ratio (HR) is the most common measure of treatment effect in clinical trials that use time-to-event outcomes such as survival. When survival curves cross over or separate only after a considerable time, the proportional hazards assumption of the Cox model is violated, and HR can be misleading. We present two measures of treatment effects for situations where the HR changes over time: the life expectancy difference (LED) and life expectancy ratio (LER). LED is the difference between mean survival times in the intervention and control arms. LER is the ratio of these two times. LED and LER can be calculated for at least two time intervals during the trial, allowing for curves where the treatment effect changes over time. The two measures are readily interpretable as absolute and relative gains or losses in life expectancy.

**Summary**

When survival curves cross over or separate only after considerable time in a trial, the hazard ratio (HR) is not an appropriate summary measure of treatment effect, because the proportional hazards assumption of the Cox model is violated and the HR changes with time
Life expectancy difference (LED) and life expectancy ratio (LER) are complementary absolute and relative measures that can be calculated for any shape of survival curves
LED is obtained by taking the difference between the mean survival times in the intervention and control arms restricted between two time points, usually the beginning of the trial and the end of follow-up; the LER is the ratio of these two quantities
LED and LER have intuitive interpretations as absolute and relative gains or losses in life expectancy due to an intervention

In randomised controlled trials (RCTs), time-to-event endpoints, such as overall survival or time to disease occurrence, are shown as Kaplan-Meier curves. The effect of an intervention compared with a control is quantified by comparing two survival curves to generate the hazard ratio (HR) and the difference in medians (when estimable), which are measures of relative and absolute effects, respectively. HR is the ratio of hazards between two treatment groups, estimated using a Cox regression model. The hazards are the instantaneous rates of occurrence of events that underlie the survival curves; for example, the instantaneous risk of death in the treatment group. This approach is only valid if the hazards are proportional to each other over time (fig 1a). The proportional hazards assumption places no constraint on the shape of one survival curve (usually the control group), only on the shape of the second (the experimental group) in relation to the other. This implies that the HR is independent of time and that the survival curves are distinct from each other and do not cross over. Median survival can be reported for any shape of curve but occurs at only one time point—it ignores the rest of the curve and can be affected by chance variability.

**Fig 1:**
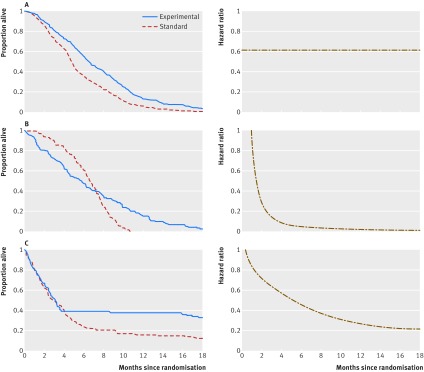
Kaplan-Meier curves with (a) proportional hazards (b) curves that cross over, and (c) curves that separate after four months. The panel of diagrams on the right hand side show how the hazard ratios change over time.

If the curves cross over or overlap for a considerable period of follow-up (more than a quarter of the follow-up period) before separating (fig 1b-c), the assumption of proportional hazards fails. This also occurs if the treatment has an early effect, where initial separation of the survival curves gets smaller or even disappears over time. HR is constant over time when the curves have proportional hazards (fig 1a), but it changes when they do not (fig 1b-c). These patterns have been observed in oncology,^1^ ^2^ ^3^ nephrology,^4^ and cardiovascular and infectious diseases^5^ ^6^ ^7^ (see supplementary figure 1). Despite HRs being misleading for non-proportional hazards, they are still the most commonly reported measure of effect, because they are readily available in all standard statistical software and are considered the standard measure for time-to-event outcomes.

An alternative recommended measure is the restricted mean survival time (RMST),^8^ which is the area under a survival curve between two time points, typically the time of randomisation and the end of the follow-up period (fig 2).^9^ The RMST of an overall survival curve is a measure of the average duration of survival over the follow-up period.^6^ ^8^ ^10^ A treatment effect can then be quantified as the difference in RMSTs or ratio of RMSTs between the experimental and control arms.

**Fig 2:**
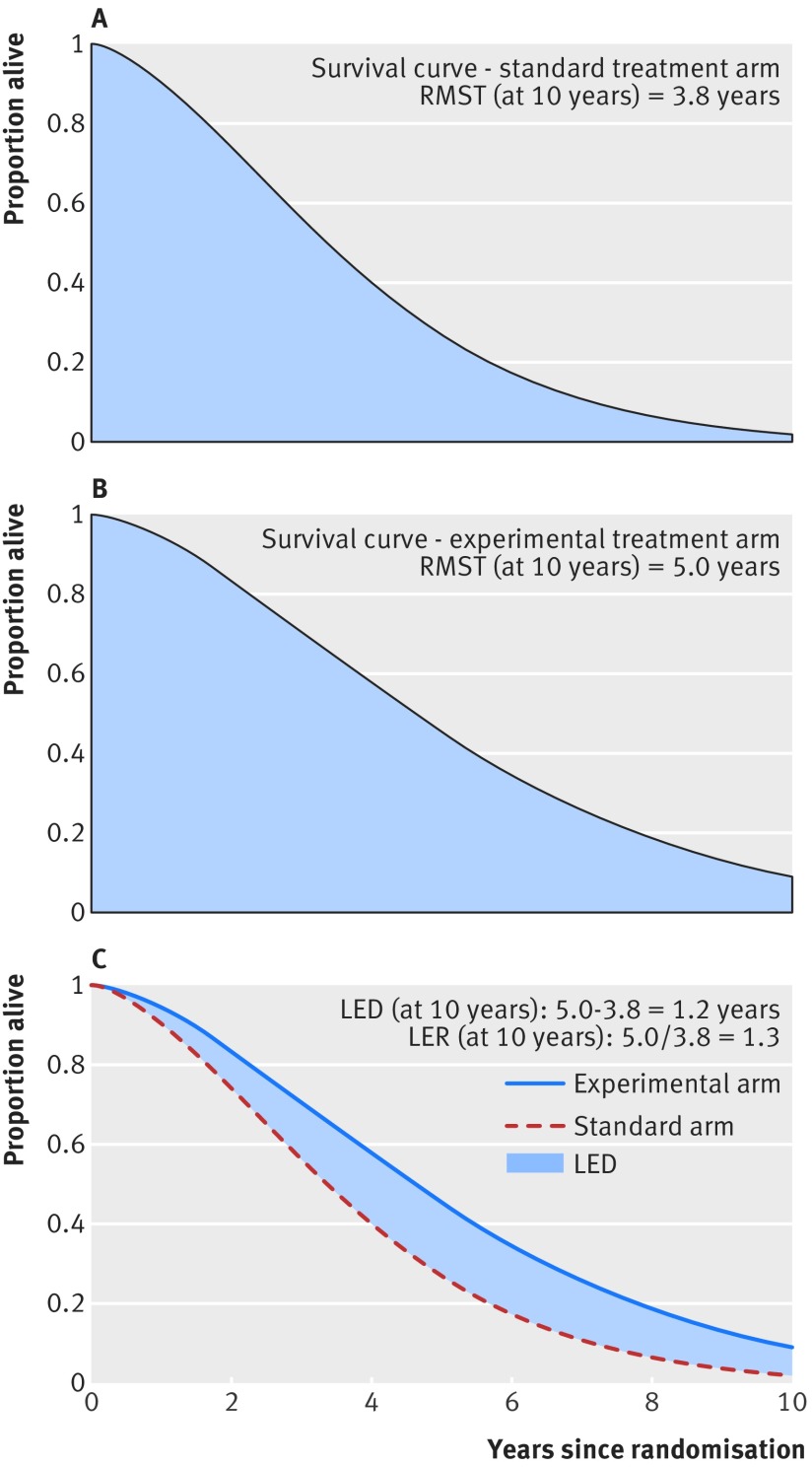
Restricted mean RMST (area under the curve) and corresponding LED and LER

## Life expectancy difference (LED) and life expectancy ratio (LER)

RMST can be calculated in four ways, which use either the Kaplan-Meier curve or a modelled curve that has been fitted to the observed data (box 1; see web extra 1 in data supplement).

Box 1 **Methods of estimating RMST**

Numerical integration of the Kaplan-Meier curve
Numerical integration of the modelled survival curve obtained using Cox regression
Numerical integration of the modelled survival curve obtained using flexible parametric modelling
Calculation of leave-one-out estimates of RMST (using any of the above methods) multiple times and taking an average

The difference between two RMSTs (experimental minus control) is called the life expectancy difference (LED). It is a measure of absolute effect, measured in units of time.^8^ Experimental RMST divided by control RMST is the life expectancy ratio (LER), a measure of relative effect.^10^ For endpoints other than overall survival, the definition of life expectancy can be changed; for example, for progression-free survival (PFS), we could use the labels LED_WP_ and LER_WP_ to indicate “without progression.”

When hazards are non-proportional, HR changes over time. Using RMST, however, allows us to calculate more than one measure of LED or LER because we can specify the time range.

Using simulation studies we estimated LED and LER for the two common forms of non-proportional hazards shown in fig 1b and fig 1c, using the four different methods for calculating an RMST (see web extra 2 for more details). Three methods had similar accuracy, of which the flexible parametric model seemed better than the others when the trial sample size was small. We provide simple STATA code to allow estimation of the LED and LER in web extra 3.

## Examples

**Fig 3:**
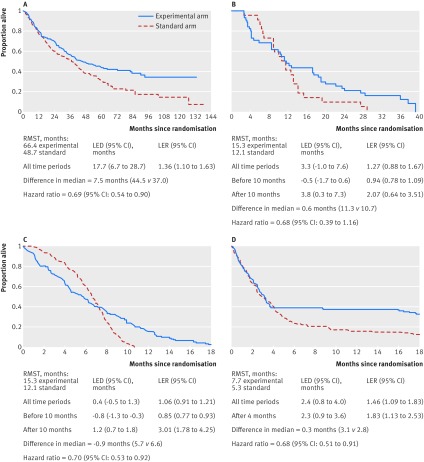
Kaplan-Meier curves that (a) diverge,^11^ (b) cross over,^12^ (c) cross over with clear harms and benefits, and (d) separate only later on in the trial. Statistical significance is indicated by a 95% CI for the LER that excludes 1 or for the LED that excludes 0.

We calculated LED and LER for three examples using a flexible parametric model (fig 3).

### Example 1: Curves that diverge

This randomised trial of 370 patients examined the addition of rituximab to standard chemotherapy for patients with untreated mantle cell lymphoma.^11^ The Grambsch-Therneau test for non-proportional hazards was statistically significant (P=0.025). Fig 3a shows the Kaplan-Meier curves for overall survival. The LER of 1.36 (95% confidence interval (CI) 1.10 to 1.63) indicates that rituximab increased life expectancy by 36% (as a relative measure). The reciprocal of 1.36 is 0.73, which is close to the HR of 0.69. This seemingly intuitive comparison should be interpreted with caution because although HR and LER both measure effect on a relative scale, they are different measures with different meanings.^10^ The difference in median survival is 7.5 months, much smaller than the LED of 17.7 months (95% CI 6.7 to 28.7), which indicates that life expectancy has improved by 17.7 months. LED is a more appropriate comparison of the survival experience between the two treatment groups. Calculations of the LED and LER are provided in web extra 1.

### Example 2: Curves that cross

The survival curves of a randomised trial investigating a drug called ADI-PEG20 in 68 patients with malignant pleural mesothelioma^12^ cross over (Grambsh-Therneau test of non-proportionality P=0.02) at approximately 10 months (fig 3b). The overall HR of 0.68 (95% CI 0.39 to 1.16) gives the false impression of benefit throughout the whole follow-up. Having a single measure of treatment effect (either HR or LER) is misleading given the shape of the curves.

We estimated the LED and LER before and after 10 months to provide a more accurate assessment of the treatment profile. Before 10 months, life expectancy with ADI-PEG20 is lower than control by 0.5 months and reduced by 6% (LER=0.94). After 10 months, the LED is 3.8 months and LER is 2.07 (but not statistically significant), indicating beneficial effects for ADI-PEG20.

Another example of curves that cross, using simulated data of 250 patients and 247 events, is shown in fig 3c. The overall LED is 0.4 months and LER is 1.06, which clearly shows how statistically significant beneficial effects seen in later months (LER is 3.01 after seven months) can be counterbalanced by statistically significant harmful effects in early months (LER is 0.85 before seven months). This results in little net effect over the whole follow-up period.

An alternative to calculating the LED and LER before and after the crossing point is to use the time at which the HR (calculated using flexible parametric survival modelling^13^) becomes <1—that is, when the experimental treatment begins to have a beneficial effect. This objective approach might minimise the appearance of arbitrarily choosing cut-off time points.

### Example 3: Curves that separate later on

Here, we used simulated data for 250 patients and 197 deaths (fig 3d). The HR of 0.68 indicates a clear benefit, but the median values are close together, producing a small difference in medians of only 0.3 months. The LER of 1.46 (95% CI 1.09 to 1.83) shows that life expectancy is increased by 46%. The LED clearly shows an absolute improvement in life expectancy (2.4 months; 95% CI 0.8 to 4.0), making it a more appropriate measure than the difference in medians. If we restrict the curves to the point after they split (at four months), the LER is noticeably higher (1.83).

The overall LED of 2.4 months may not seem striking because it takes into account that many patients do not benefit before four months. After four months, the LED is similar (2.3 months), because it reflects a smaller area under the Kaplan-Meier curve—that is, life expectancy (compared with the whole follow-up period).

In scenarios like this, only some patients benefit (potentially a minority). Investigators should examine the proportion of patients who are at risk until the curves split and consider the cost of treating them (with side effects) to be balanced against the proportion who would (significantly) benefit.

Curves like this have been seen in cancer trials of immunotherapy. In one such trial—pembrolizumab versus chemotherapy for treating relapsed melanoma^14^—the HR was 0.57 (a strong relative effect) but the absolute improvement was small, with a median PFS of 2.9 versus 2.7 months. The RMSTs were 5.4 versus 3.6 months, producing an LED of 1.8 months and LER of 1.5, which better reflect the overall treatment effect.

## Discussion

Kaplan-Meier curves from RCTs exhibiting non-proportional hazards are seen in the literature,^10^ ^15^ ^16^ but relatively few investigators tackle this problem in their reports, and fewer still take it into account when estimating treatment effects. Non-proportionality could be caused by the mechanism of action of a treatment, supported by biological evidence; a biomarker, where the Kaplan-Meier curves do have proportional hazards when analysed separately according to concentrations of the marker^2^; or treatments with long term benefits but high early mortality. In other cases, it might simply be a chance finding in a small trial or influenced by the trial design.

In some immunotherapy trials for advanced cancer, no difference is seen in median PFS.^1^ On closer inspection, the timing of the first radiological assessment scan for progression coincides with the median PFS, indicating that progression actually occurred before this time in many patients; the timing of the first scan is too late. Interpreting these results requires careful scrutiny, regardless of the measure of treatment effect.

Our examples show that HRs and differences in median times can be misleading when the curves do not have proportional hazards. They can underestimate the absolute treatment benefit (example 2) or overestimate the relative effect (example 3). In these scenarios, LED and LER are more appropriate measures of effect. Using survival (event) rates at a single time point might avoid the problem of non-proportional hazards, but because LED and LER are derived using the entire curves, they give a more reliable estimate of treatment efficacy than one based on a single time point, which is more likely to be influenced by chance variability.

When the treatment effects clearly change over time (examples 2 and 3), no single “average” estimate of treatment efficacy can effectively show this, whether using HR, LED, or LER. Researchers should acknowledge the limitation of using overall measures of effect. In such cases LED and LER can be estimated for specific parts of the curves; for example, before and after they cross over or after they separate. This is similar in principle to splitting the time axis into several periods and estimating the HR in each using a time dependent Cox regression model, but LED and LER can be calculated for any shape of curve, even within each time interval.

We recommend reporting two LED and LER estimates to distinguish early from late treatment effects. Alternatively LED and LER can be plotted according to follow-up time. Researchers should try to understand and explain the biological or mechanistic reasons behind such patterns (analogous to exploring heterogeneity in meta-analyses). Specifying the time periods before the trial begins is preferable, ideally those with clinical relevance, to avoid focusing on parts of the curves where the treatment effect happens to be the largest, although in our examples the time points would not be known until the data are visualised. LED and LER could be calculated at multiple time points and corrected for multiple testing, which has recently been proposed, though this may make the interpretation of a trial more complex.^9^ Finally, LED and LER can always be estimated, even when the median times have not yet been reached.

## Recommendations

When HR changes with time, we recommend using LED and LER as absolute and relative measures of treatment effect. These measures are clinically meaningful and simple to understand. When survival curves cross over, LED and LER can be estimated for specific parts of the curves—for example, before and after the curves cross over—to distinguish between early and late treatment effects.

## Web extra

Extra material supplied by authors

**Supplementary file:** Web extras

## Notes

Contributors: The initial concept of using both the difference and ratio of RMSTs (instead of median difference and hazard ratio) in trials with non-proportional hazards came from AH and HMD. AH further developed the concept and designed the analyses. HMD created the names of the two measures. HMD, PR, and AH drafted the article and approved the final version. AH is guarantor. All authors take responsibility for the integrity of the data and the accuracy of the data analysis. HMD, PR, and AH are medical statisticians working in cancer clinical trials. PR has worked for many years on methods to analyse trials with non-proportional hazards and has published extensively in statistical journals.
