# Supplementary material for: Life expectancy difference and life expectancy ratio: two measures of treatment effects in randomised trials with non-proportional hazards
Source: BMJ. 2017 May 25;357:j2250. doi: 10.1136/bmj.j2250 (PMC5444092; doi:10.1136/bmj.j2250)
Supplement: Supplementary file 1 — Supplementary file: Web extras [file dehh036921.ww1.pdf]

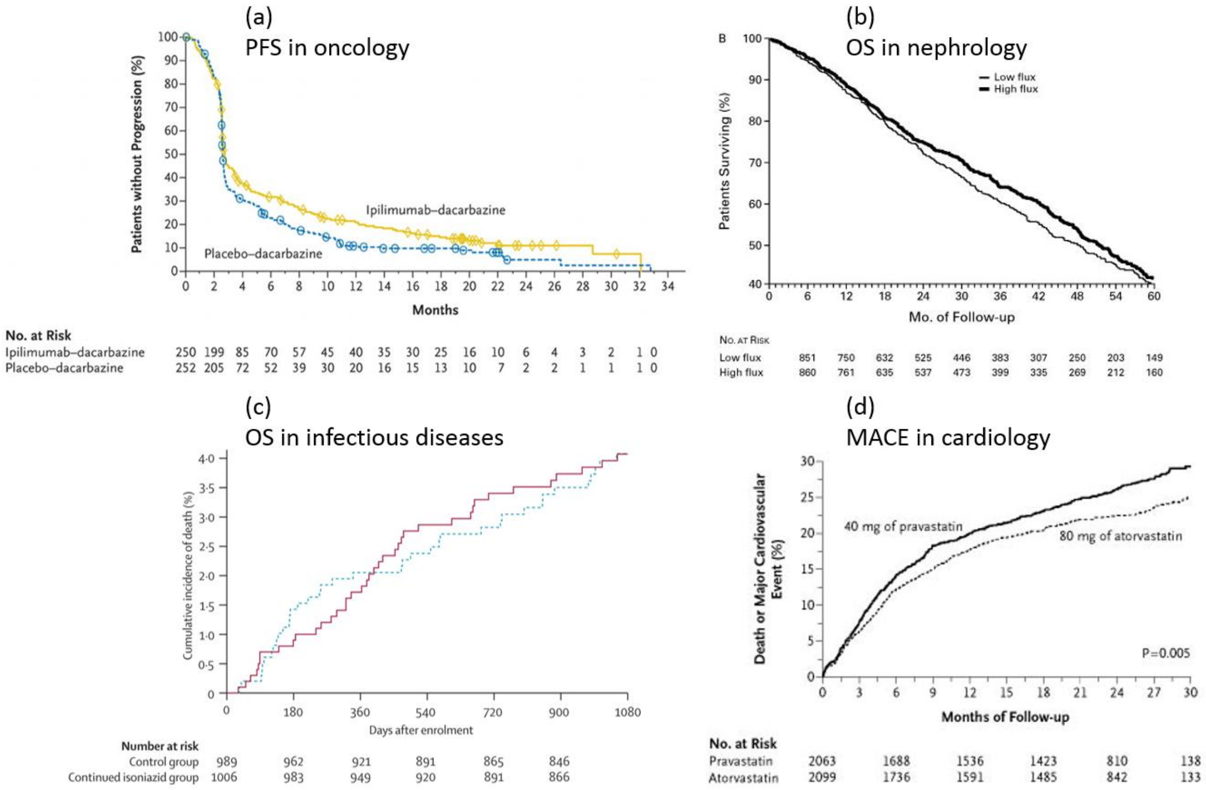

Web Figure 1. (a) progression-free survival (FPS) in oncology trial[1], (b) overall survival (OS) in nephrology trial[4], (c) OS in infectious diseases trial[7], (d) death or major adverse cardiovascular event (MACE) in cardiology trial[5].

Web extra 1: Calculating a restricted mean survival time (RMST)

There are four methods for estimating an RMST (where T_{end} is the time point up to which the area of the curve is required):

1. the area under the Kaplan-Meier (KM) curve, obtained by numerical integration using standard algebra up to T_{end}
2. fitting a Cox regression model through the observed data values, then the RMSTs are obtained again by integration but using the modelled curve (the problem of this approach is that the regression modelling assumes proportional hazards to produce the fitted curve).
3. fitting a flexible parametric survival model (in which the log of the cumulative hazard of the control arm is modelled as a restricted cubic spline in log time[13]), the curve is estimated and then integrated up to T_{end} to get the RMST.
4. using leave-one-out estimates, where RMSTs are calculated multiple times using any of the above methods of finding the area under the curve, each observation is excluded in turn, then a form of average is taken[17]

To obtain 95% confidence intervals of the LED and LER we first obtain the standard error (SE) of the RMSTs ($SE_{RMSTexp}$ refers to the SE of the RMST in the experimental arm, $SE_{RMSTcont}$ refers to the RMST of the control arm), then the interval is LED (or LER) ±1.96 x SE. The standard errors of LED and LER (using Taylor's expansion) are obtained using the following formulas:

$$SE\ (LED) = \sqrt{{SE_{RMSTexp}}^2 + {SE_{RMSTcont}}^2}$$

$$SE\ (LER) \approx \sqrt{\left\{\frac{RMSTexp}{RMSTcont}\right\}^2 * \left\{\frac{{SE_{RMSTexp}}^2}{RMSTexp^2} + \frac{{SE_{RMSTcont}}^2}{RMSTcont^2}\right\}}$$

Standard statistical softwares can compute an RMST and its standard error (SE). The leave-one-out technique is implemented in R [18], SAS [18] and STATA [19]. Flexible parametric models are specified in STATA [20], and also in R (see https://cran.r-project.org/web/packages/rstpm2/rstpm2.pdf). The STATA commands strmst and strmst2 provide integration solutions to compute RMST.

In example 1, the STATA outputs are:

```
. tab rmst138
```

Prediction	Freq.	Percent	Cum.
48.7487	184	49.73	49.73
66.43575	186	50.27	100.00
Total	370	100.00	

```
. tab rmst138_se
```

Standard error: rmst138	Freq.	Percent	Cum.
3.635276	184	49.73	49.73
4.288769	186	50.27	100.00
Total	370	100.00	

The RMST of the experimental and control arms are 66.4 and 48.7 respectively. The LED is therefore calculated as:

$$LED = 66.4 - 48.7 = 17.7$$

The SE of the experimental and control arms are 4.3 and 3.6 respectively. The SE of the LED is obtained as:

$$SE\ (LED) = \sqrt{4.3^2 + 3.6^2} = 5.6$$

$$95\%\ CI\ for\ LED = 17.7 \pm 1.96 * 5.6 = 6.7\ to\ 28.7$$

The LER is obtained by dividing the RMSTs:

$$LER = \frac{66.4}{48.7} = 1.36$$

The SE of the LER is obtained as:

$$SE\ (LER) \approx \sqrt{\left\{\frac{66.4}{48.7}\right\}^2 * \left\{\frac{4.3^2}{66.4^2} + \frac{3.6^2}{48.7^2}\right\}} = 0.13$$

$$95\%\ CI\ for\ LER = 1.36 \pm 1.96 * 0.13 = 1.10\ to\ 1.63$$

For references, see:

1/ Cronin, A., L. Tian, and H. Uno, strmst2 and strmst2pw: New commands to compare survival curves using the restricted mean survival time. Stata Journal, 2016. 16(3): p. 702-716.
2/Royston, P., Estimating the treatment effect in a clinical trial using difference in restricted mean survival time. Stata Journal, 2015. 15(4): p. 1098-1117.

Web extra 2: Estimating the LER

We examined the four methods of calculating RMST (specified in Web extra 1), to see how well they estimate the Life Expectancy Ratio. This was achieved using a simulation study in which the true survival curves were of known shape, using the two common forms of non-proportional hazards as shown in Figures 1b-c (i.e. where curves cross each other, or where the curves lie close together for several months/years and only then separate). We also used two trial sample sizes (n=100 and n=500).

For the flexible parametric model, we used three different specifications: we assigned 3, 5 or 10 degrees of freedom (d.f.) to the baseline function and one d.f. to the time-dependent treatment effect, as recommended in [8]. Other choices of d.f. can be made in practice, for example up to 5 d.f. for the time-dependent effect.

We had 24 different scenarios (6 methods x 2 forms of non-proportional hazards x 2 sample sizes). We replicated the simulations 1000 times in each of the 24 scenarios. We used the Stata command survsim to simulate the survival curves using a Gompertz distribution.

In the Table below, we evaluated the different methods using bias and mean squared error (MSE) with respect to the LER. Bias measures the difference between the estimated LER and its true value, and the smaller the bias, the more reliable the model. The MSE measures the square of the difference between the estimated LER and its true value. It incorporates both the bias and the variance of the estimated LER, and therefore is a complement to the bias. The variance is important to consider as an estimator can be unbiased on average but with a very large variance. The smaller the MSE the greater the accuracy.

As expected, the Cox model had the worst performance (unsurprising given that it assumes proportional hazards when fitting the curves, before an RMST is calculated). The other methods all had acceptable performance, though the flexible parametric approach was better for the trial size of n=100. We also examined the LED in the simulations, and reached the same conclusions.

Web table: Results of the simulations for LER, using the four estimation techniques: Cox, KM, leave-one-out (LOO) and flexible parametric (Flex), for the two forms of non-proportional hazards and two sample sizes

	Cox	KM	LOO	Flex (3 df)	Flex (5 df)	Flex (10 df)
Survival curves crossing						
n = 100						
Bias	0.1584	-0.0102	0.0508	0.0184	0.0216	0.0200
MSE	0.0368	0.0123	0.0111	0.0132	0.0134	0.0133
n = 500						
Bias	0.1639	-0.0014	0.0031	0.0190	0.0204	0.0205
MSE	0.0292	0.0024	0.0022	0.0029	0.0030	0.0030
Survival curves separating						
n = 100						
Bias	0.1106	0.0106	0.0042	-0.0019	-0.0021	-0.0163
MSE	0.0158	0.0050	0.0046	0.0049	0.0045	0.0054
n = 500						
Bias	0.0936	0.0003	-0.0009	0.0167	0.0167	0.0169
MSE	0.0094	0.0010	0.0010	0.0013	0.0013	0.0013

Web extra 3: STATA code to calculate the LER and LED

```
*****************************************************************
* install packages
*****************************************************************
ssc install stpm2
ssc install rcsgen
ssc install survsim
ssc install moremata

*****************************************************************
* load the dataset
** treatment variable coded as 0/1 (0 for  control arm, 1 for experimental arm)
** stime variable in units of time and >=0
** event variable coded as 0/1 (1 for event, 0 for censoring)
*****************************************************************
* visualize KM curves
sts graph, by(treatment)

*****************************************************************
* calculate the RMSTs using flexible
* parametric modelling with 3 d.f. for the  baseline hazard and
* 1 df for the time dependency
*****************************************************************
* fit model
stpm2 treatment, df(3) dftvc(1) tvc(treatment) scale(haz)
* calculate RMST and SEs
predict rmst, rmst tmax(10) stdp // adjust tmax depending on study
* RMST for control arm
summarize rmst if treatment==0
scalar rmst_S0=r(mean)
summarize rmst_se if treatment==0
scalar SE_rmst_S0=r(mean)
* RMST for experimental group
summarize rmst if treatment==1
scalar rmst_S1=r(mean)
summarize rmst_se if treatment==1
scalar SE_rmst_S1=r(mean)

* calculate difference of RMSTs, giving the LED
scalar LED = rmst_S1-rmst_S0
display LED // this is the LED

scalar se_LED = (SE_rmst_S0^2+SE_rmst_S1^2)^0.5
scalar ci_up_LED = LEG + 1.96*se_LEG
scalar ci_low_LED = LEG - 1.96*se_LEG
display ci_up_LED  // this is the upper bound of the 95% CI
display ci_low_LED // this is the lower bound of the 95% CI

* calculate ratio of RMSTs, giving the LER
scalar LER = rmst_S1/rmst_S0
display LER // this is the LER
```

```
scalar se_LER = ( (rmst_S1/rmst_S0)^2 * ((SE_rmst_S1/rmst_S1)^2 + (SE_rmst_S0/rmst_S0)^2) )^0.5
scalar ci_up_LER = LER + 1.96*se_LER
scalar ci_low_LER = LER - 1.96*se_LER
display ci_up_LER  // this is the upper bound of the 95% CI
display ci_low_LER // this is the lower bound of the 95% CI
```
